# Detection of Microbehavior Intervals for Predicting Mental Health: Clinically Relevant and Advanced Multimodal Temporal Analysis

**DOI:** 10.2196/87049

**Published:** 2026-05-27

**Authors:** Sapir Gershov, Charlotte E Hilberdink, Yiwen Zhao, Sarah B Birnbaum, Victoria Mueller, Stephen P Wall, Katharina Schultebraucks

**Affiliations:** 1 Department of Psychiatry Grossman School of Medicine New York University New York, NY United States; 2 Department of Biomedical Engineering Tandon School of Engineering New York University New York, NY United States; 3 Department of Emergency Medicine Grossman School of Medicine New York University New York, NY United States; 4 Department of Population Health Grossman School of Medicine New York University New York, NY United States

**Keywords:** burnout, posttraumatic stress disorder, PTSD, anomaly detection, multimodal, health care workers

## Abstract

**Background:**

Health care workers (HCWs) face sustained psychological demands that place them at heightened risk for burnout and posttraumatic stress disorder (PTSD). However, assessing psychological distress in this population remains challenging because of stigma, underreporting, and the limitations of self-report tools. Although nonverbal behaviors such as facial expressions and gaze hold diagnostic promise, most approaches overlook the fine-grained, temporal fluctuations in these signals. In this study, we focused on microbehavior intervals—brief, involuntary changes in multimodal nonverbal signals—that emerge during emotion-eliciting interviews.

**Objective:**

This study aimed to determine whether microbehavior intervals improve the discrimination of psychological distress profiles among HCWs with symptoms of burnout and PTSD.

**Methods:**

HCWs participated in a semistructured interview that included 5 work-related, emotionally charged questions and that was recorded via Webex (online video platform). Participants also completed validated questionnaires for burnout (Maslach Burnout Inventory General Survey 9-item) and PTSD (PTSD checklist for *Diagnostic and Statistical Manual, 5th edition*). Recordings were analyzed with computer vision models to generate time-series data of facial expressions, head movement, gaze, body posture, and hand gestures. An unsupervised anomaly detection model (MOMENT [a Family of Open Time-Series Foundation Models]) isolated microbehavior intervals without requiring manual labels. Features derived from these intervals were used to train a deep learning classifier that predicted 4 symptom classes of psychological distress: “moderate-severe burnout,” “subthreshold-provisional PTSD,” “burnout+PTSD,” and “resilient.” We conducted an ablation study by systematically removing one behavioral data stream at a time. Finally, we conducted an explainability analysis to characterize the features driving model predictions.

**Results:**

We analyzed 258 interview recordings from 151 HCWs. Per interview, an average of 19.65 (SD 6.01) microbehavior intervals were detected, each lasting an average of 1.31 (SD 1.10) seconds. The classifier demonstrated robust performance across classes, achieving a macro– *F*_1_-score of 0.75 and a macro area under the receiver operating characteristic curve of 0.80 on held-out data. Ablation analysis showed that excluding gaze or arousal-valence signals caused the largest performance declines, particularly in recall and *F*_1_-score. The explainability analysis revealed distinct temporal patterns across symptom classes, with irregularity and variability in microbehaviors emerging as key predictors.

**Conclusions:**

Focusing on microbehavior intervals yields a scalable, interpretable, and annotation-free framework for detecting psychological distress from nonverbal signals. By moving from whole-video features to fine-grained multimodal temporal modeling, we successfully captured subtle, involuntary fluctuations in nonverbal responses to emotion-eliciting questions. This multimodal approach enables an objective, robust, and explainable assessment of psychological distress and offers a promising complement to conventional psychometric assessments.

## Introduction

Frontline professionals operating in high-stakes environments—such as health care workers (HCWs) [1], military personnel [2], and first responders [3]—are frequently exposed to emotionally intense, cognitively demanding, and potentially traumatic events. These chronic occupational stressors place them at elevated risk for burnout and posttraumatic stress disorder (PTSD) [4], which frequently co-occur and exacerbate psychological burden [5,6].

Burnout is an occupational syndrome characterized by exhaustion and disengagement [7], and it affects approximately one-third to one-half of HCWs [8-10]. PTSD, in contrast, is a trauma-related disorder defined by intrusive memories, hyperarousal, and avoidance [11], and symptoms are reported by up to 1 in 5 trauma-exposed HCWs [12,13]. Despite this burden, assessing symptom severity remains a major challenge due to stigma, underreporting, emotion suppression, and the limitations of traditional self-report tools [3,14,15]. These barriers highlight the need for complementary methods that can detect psychological distress without relying solely on verbal disclosure or self-report questionnaires. 

Nonverbal cues offer a valuable, often underused dimension for psychiatric assessments, particularly in individuals who are unaware of or suppress their emotional states [16,17]. Among these cues, facial microexpressions, which represent brief, spontaneous, and involuntary muscle movements that are difficult to consciously regulate, have garnered substantial attention as potential reliable and objective indicators of internal affect [18-20]. Recent machine learning studies have demonstrated that subtle irregularities in the temporal patterns of microexpressions are strongly associated with individuals with depression [16,21], highlighting their potential as objective markers for mental health assessment. However, the clinical utility of microexpressions remains limited by several challenges, including: (1) data acquisition using high-frame-rate cameras (100-200 frames per second), (2) the need for highly sensitive algorithms, (3) time-intensive annotation, and (4) substantial interrater variability even among expert annotators [20].

Recent evidence suggests that multimodal machine learning frameworks, which analyze video-recorded interviews using computer vision and natural language processing, may offer an alternative methodology for screening for psychological disorders [22-25]. Although promising, many of these approaches reduce dynamic behavior to static average features [22] or extract only sentence-level features [26], thereby overlooking the fine-grained temporal fluctuations in verbal and nonverbal cues. By collapsing rich behavioral dynamics into summary statistics, these methods risk omitting subtle yet meaningful signals that may carry critical diagnostic information [17,27-29].

Although facial microexpressions have traditionally served as the canonical example of emotionally revealing nonverbal behavior, the underlying mechanism—involuntary expression of emotional states through brief, spontaneous motor acts—is shared across many other nonverbal behavior modalities. In the context of burnout and trauma-related disorders, such involuntary expressions can manifest as blunted or exaggerated facial expressivity [30], disrupted eye contact [31,32], tense or withdrawn posture [33], and restlessness [34,35]. This broader framing moves beyond face-centric paradigms, allowing us to examine a more holistic perspective of nonverbal behaviors as high-resolution, interpretable signals of internal distress.

Spontaneous changes in nonverbal behavior signals, termed microbehaviors, can be modeled in the same way as microexpressions. Specifically, we can temporally localize fluctuations and changes in body movement that arise spontaneously in response to a stimulus. Microbehaviors manifest as sparse spikes within continuous nonverbal signals and exhibit distinct onset, apex, and offset phases. When treated as a time-series signal, microbehaviors can be segmented and interpreted using time-series analytical techniques such as anomaly detection [36]. Our hypothesis is that abrupt fluctuations in multimodal nonverbal behaviors may reflect emotional dysregulation and that, by quantifying their spatiotemporal changes, we can use these signals as objective digital biomarkers of psychological distress.

To test this hypothesis, we developed an unsupervised multimodal video analysis framework that captures microbehaviors using multivariate time-series modeling and zero-shot anomaly detection. Our framework focuses exclusively on facial expression, head movement, gaze, body posture, and hand movement—nonverbal modalities consistently linked to burnout [32] and PTSD [24,31,33]. Although speech-based and linguistic features have shown promise in prior work [24,37-39], we intentionally excluded them from our framework because their interpretation depends on language-specific models that may not generalize across cultural contexts, can be biased by differences in language fluency, and may be influenced by an individual’s comfort with verbal self-disclosure [40]. By contrast, nonverbal behaviors are considered more language-independent and culturally robust, thus making them more suitable for detecting psychological distress [41-43].

In summary, this study introduces a novel multimodal framework that identifies brief periods of heightened behavioral expressivity and evaluates whether temporal features derived from these intervals can predict burnout and PTSD symptom profiles among HCWs.

## Methods

### Participants

Participants were drawn from “Early Signs: digital phenotyping to identify digital biomarkers for predicting burnout and cognitive functioning in emergency department clinicians,” an ongoing National Institutes of Health (NIH)–funded longitudinal study (R01HL156134) examining burnout and well-being among HCWs in emergency and trauma care. Inclusion criteria required participants to be aged ≥18 years, fluent in English, and engaged in direct patient contact with a typical full-time clinical schedule (including individuals who were temporarily working part-time due to burnout or leave). Medical students and temporary staff were not eligible. Recruitment was conducted among emergency and trauma clinicians and emergency department staff working within major health systems across the New York City metropolitan area. Outreach efforts combined targeted online advertisement (eg, Facebook [Meta Platforms Inc] and iConnect) with structured in-person engagement by trained research staff. Interested individuals were directed to an institutional review board–approved key information sheet and study contact information. Research staff subsequently followed a standardized workflow to verify eligibility, address questions, and obtain written informed consent before scheduling an in-person assessment. Recruitment settings and procedures were standardized across participating sites.

As part of this study, participants were scheduled for semistructured interviews, which were recorded via an institution-approved, HIPAA (Health Insurance Portability and Accountability Act)-compliant secure video platform (Webex; Cisco Systems Inc). The interview included questions regarding work-related events and stressful experiences. In addition, participants completed self-reported questionnaires regarding their current psychological symptom levels for burnout and PTSD.

### Ethical Considerations

The study protocol was reviewed and approved by the institutional review board of New York University Grossman School of Medicine (i23-00060) and was conducted in accordance with the Declaration of Helsinki and the US Federal Policy for the Protection of Human Subjects (Common Rule, 45 CFR 46 [44]). All participants provided written informed consent before participation. Participants were informed that participation was voluntary, that they could decline to answer any question or withdraw from the study at any time, and that their decision to participate or withdraw would not affect their employment, salary, benefits, clinical care, or performance evaluations.

To protect participant privacy and confidentiality, interviews were recorded using an institution-approved, HIPAA-compliant secure video platform (Webex). Because video and audio recordings may contain identifiable information, recordings and related study data were stored on secure institutional systems, labeled with study identifiers when possible, and accessible only to authorized study personnel. Identifying information was not included in the analytic datasets used for this manuscript, and no identifiable participant images, audio, video, or direct quotations are presented in the article or supplementary materials.

Participants were compensated for their time and reimbursed for travel-related costs associated with study participation. Compensation was provided according to the institutional review board–approved study protocol and was not contingent on completion of all study procedures.

### Semistructured Interview

The interview included 5 open-ended questions targeting emotionally evocative situations, such as adverse patient outcomes and future expectations. During the recording of the interview, only the participant’s camera was turned on, which allowed us to capture their spontaneous, nonverbal responses with minimal interference. This design aimed to naturally evoke emotional and cognitive states relevant to burnout and trauma-related symptoms while preserving the conversational flow and ecological validity of real-world interactions. The interview questions are provided in the Multimedia Appendix 1.

### Psychometric Assessments

Burnout symptoms were assessed using the abbreviated Maslach Burnout Inventory General Survey 9-item [45], which includes 9 items across 3 subscales: emotional exhaustion, depersonalization, and personal accomplishment. Following Rotenstein et al [46], participants were labeled as presenting “severe” symptoms if they met the following thresholds: personal accomplishment ≤12, emotional exhaustion ≥11, and depersonalization ≥7. Those who met just 2 of the 3 thresholds were labeled as “moderate.” Participants who did not meet the criteria for “severe” or “moderate” were labeled as “no burnout.”

PTSD symptoms were measured using the PTSD checklist for *Diagnostic and Statistical Manual, 5th edition* [47], a 20-item scale based on *Diagnostic and Statistical Manual, 5th edition* criteria. According to Klein et al [48] and Biscoe et al [49], participants were labeled as “provisional PTSD” if they met the diagnostic threshold across all 4 symptom clusters or had a total score of ≥31. Those meeting only 3 clusters and scoring <31 were labeled as “subthreshold PTSD.” Participants who did not meet the criteria for “provisional PTSD” or “subthreshold PTSD” were labeled as “no PTSD.”

On the basis of their symptomatology, participants were divided into the following 4 classes: “resilient,” comprising participants who met neither burnout nor PTSD symptoms criteria; “moderate-severe burnout,” comprising participants who met only “moderate” or “severe” burnout symptoms criteria; “subthreshold-provisional PTSD,” comprising participants who met only “provisional” or “subthreshold” PTSD symptoms criteria; and “burnout+PTSD,” comprising participants who met criteria for both “moderate-severe burnout” and “subthreshold-provisional PTSD.”

### End-to-End Framework Construction

#### Overview

**Figure 1:**
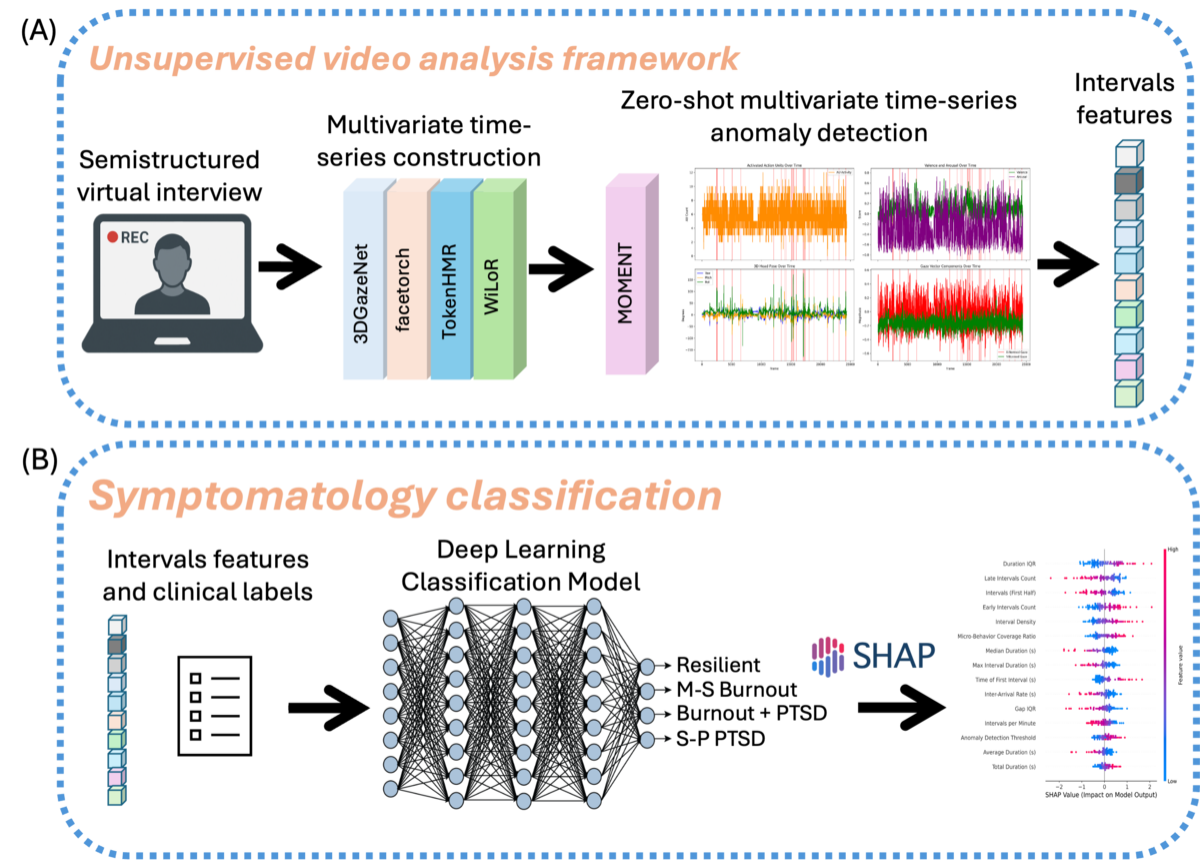
Overview of the proposed framework. The pipeline is divided into 2 components: (A) an unsupervised video analysis framework that extracts multivariate time-series data from gaze direction (3DGazeNet), facial activity (facetorch), body movement (TokenHMR), and hand movement (WiLoR; microbehavior intervals are detected via zero-shot anomaly detection (MOMENT [a Family of Open Time-Series Foundation Models]) and are then analyzed to construct interval features) and (B) a symptomatology classification component in which features, derived from intervals, are used to train a deep learning model that predicts symptomatology classes derived from self-report psychological measures (model interpretability is assessed using SHAP [Shapley Additive Explanation], which highlights features contribution to each class). PTSD: posttraumatic stress disorder.

Figure 1 illustrates the complete end-to-end architecture of our framework, encompassing (1) video signal processing, (2) anomaly detection, (3) feature extraction, and (4) classification model training and analysis. All processes were executed on a single NVIDIA A100 Tensor Core GPU (NVIDIA Corp) using Python (version 3.10), PyTorch (version 2.0.0), and scikit-learn (version 1.6.1). We followed best-practice reporting guidelines for prediction modeling and machine learning in health research (Transparent Reporting of a Multivariable Prediction Model for Individual Prognosis or Diagnosis–artificial intelligence [50] and Prediction Model Risk of Bias Assessment Tool–artificial intelligence [51]).

#### Video Acquisition and Preprocessing

Assessments were recorded remotely using the Webex platform and a standard webcam, producing videos at approximately 25 frames per second and 720p to 1080p resolution. All recordings were compressed using the Moving Picture Experts Group-4 format, and the full interview video was processed without any content-based manual trimming. Because webcam recordings exhibited occasional frame drops, we relied on decoded time stamps to maintain temporal ordering.

#### Multimodal Nonverbal Behavioral Time Series

To capture dynamic nonverbal signals indicative of psychological distress, we implemented a multimodal framework that processes raw video-recorded interviews and constructs comprehensive time series that are both multimodal and multivariate. Each video was processed frame-by-frame using state-of-the-art computer vision models, resulting in the following behavioral time series:

- Facial expressions: we used “facetorch” [52], a modular affective computing framework, which provided (1) binary activation of facial action units, encoding fine-grained facial muscle movements, and (2) continuous valence and arousal estimates based on facial expressions.
- 3D head pose estimation: we extracted 3D head orientation (yaw, pitch, and roll) using the “facetorch” pose estimation module.
- 3D gaze estimation: we used “3DGazeNet” [53] to estimate participants’ 3D gaze direction. This model was selected for its robustness to head pose and lighting variations, making it suitable for naturalistic, webcam-based recordings.
- 3D body pose estimation: we used “TokenHMR” [54], which estimates 3D human body pose based on predefined key points in a temporally consistent manner. For each frame, we extracted the 3D coordinates of upper-body key points and quantified gross motor activity as the mean Euclidean distance (displacement) between key points across consecutive frames, yielding a per-frame movement-magnitude time series.
- 3D hand pose estimation: fine-grained hand movements were estimated using “WiLoR” [55], a 3D hand localization and mesh reconstruction model. This provided us with 3D key points for both hands, allowing us to track gestural patterns.

Frames where a target signal was not observable (eg, face or hands out of frame) were retained in the sequence and coded as missing outputs. All modalities were synchronized to ensure temporal alignment. For each frame, the extracted time series were concatenated to form a multivariate (multichannel) representation that preserves the fine-grained temporal structure of the recorded nonverbal behavior.

#### Anomaly Detection of Microbehavior

To identify microbehavior intervals throughout the interview recording, we applied an anomaly detection to the multivariate time series. Anomaly detection models define “normal” time-series patterns that conform to their learned expectations of smoothness, predictability, and local temporal structure. Deviations from these expectations—such as abrupt changes, irregular bursts, or unexpected fluctuations—are interpreted as “anomalies,” and represented as numerical anomaly scores that quantify the extent to which a data point deviates from what is considered normal within a sequence of data points collected over time. Higher anomaly scores indicate a greater likelihood of a data point being an anomaly.

In our framework, we used MOMENT (a Family of Open Time-Series Foundation Models) [56], which is a foundation model trained on diverse time-series tasks, selected for its ability to detect outlier patterns in a zero-shot manner without requiring labeled data or domain-specific fine-tuning. MOMENT anomaly scores quantify reconstruction-based deviations from expected behavior and are computed independently for each univariate signal channel (ie, nonverbal behavior) at each time point. These scores vary in scale, depending on the input signal, in which higher values indicate a more behaviorally or contextually abnormal pattern within the observed sequence. A frame-level total anomaly score was obtained by summing the anomaly scores across nonverbal behaviors. Microbehavior segments were defined as contiguous windows in which the frame-level total anomaly score exceeded the 95th percentile threshold. Finally, on the basis of empirical observation, segments separated by <1 second were merged to form discrete microbehavior intervals. We verified that this rule primarily reduced overfragmentation (often caused by transient tracking noise or brief occlusions) without materially changing the overall interval distribution. This gap-bridging step is consistent with prior work in microexpression and frame-based affect modeling, where expressions are inherently brief (often subsecond) and continuous runs of frames are treated as a single event rather than multiple occurrences [16,21].

It is important to note that no human annotations or manual coding were used to identify anomalous frames or intervals; microbehavior intervals were derived solely from MOMENT anomaly scores using the fixed percentile-thresholding and merging rules described previously.

#### Interval Feature Extraction

After the segmentation procedure, for each video recording, we extracted 36 interval-level statistical and temporal features that summarize the microbehavior intervals (eg, number of intervals, average duration, density, variability). These interval features were computed manually using a custom Python implementation.

#### Classification Model Training and Explainability

The final part of the framework involved training a deep learning classification model using the extracted features of the microbehavior intervals to predict the 4 symptom classes (“resilient,” “moderate-severe burnout,” “subthreshold-provisional PTSD,” and “burnout+PTSD”).

A custom classification model, a feedforward fully connected multilayer perceptron, was constructed and optimized using Optuna (version 4.8.0) [57]. The full dataset was split into a training set (80%) and an independent held-out test set (20%), using stratified sampling to preserve class distribution. Model training and hyperparameter fine-tuning were performed using stratified 5-fold cross-validation with cross-entropy loss and macro–*F*_1_-score as the objective metric. To avoid overfitting, we also initiated early stopping based on validation loss. To prevent information leakage, all splits (training and test sets, as well as cross-validation folds) were grouped by participant, ensuring that recordings from the same HCW did not appear in both the training and evaluation sets.

**Figure 2:**
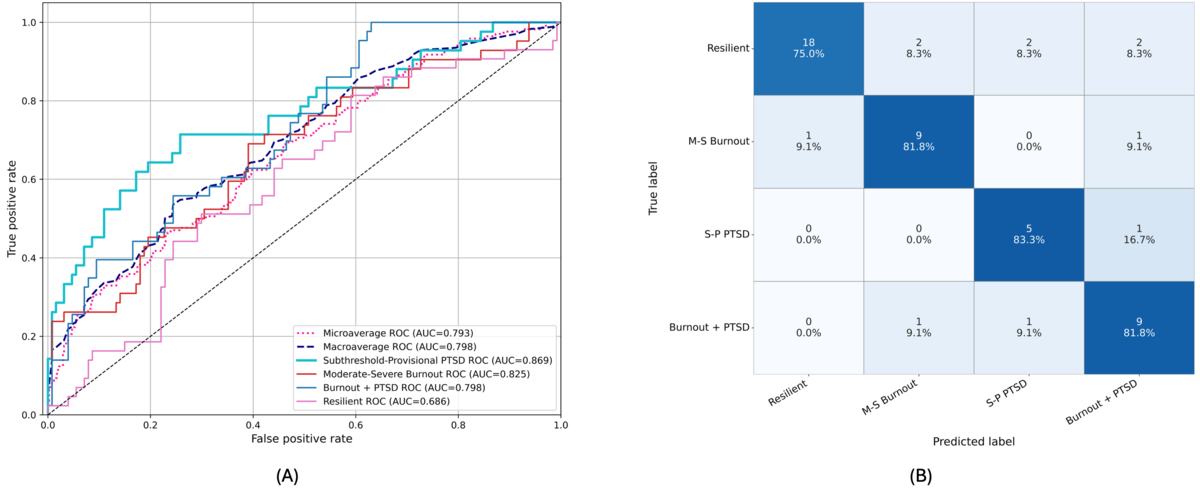
Model performance across the 4 psychological comorbidity profiles. (A) One-vs-rest receiver operating characteristic (ROC) curves on the independent held-out test set and (B) row-normalized confusion matrix for the held-out test set, with counts shown within each cell. AUC: area under the curve; M-S: moderate-severe; PTSD: posttraumatic stress disorder; S-P: subthreshold-provisional.

To address the imbalance of symptom classes within our dataset, the Synthetic Minority Oversampling Technique [58] was applied within each training fold. Model performance was assessed on the held-out test set using weighted accuracy, precision, recall, and macro– *F*_1_-score, as well as macro, micro, and per-class area under the receiver operating characteristic curve scores (Figure 2). Additionally, we evaluated the influence of different modalities on model performance by conducting an ablation study (Figure S4 in the Multimedia Appendix 1).

**Figure 3:**
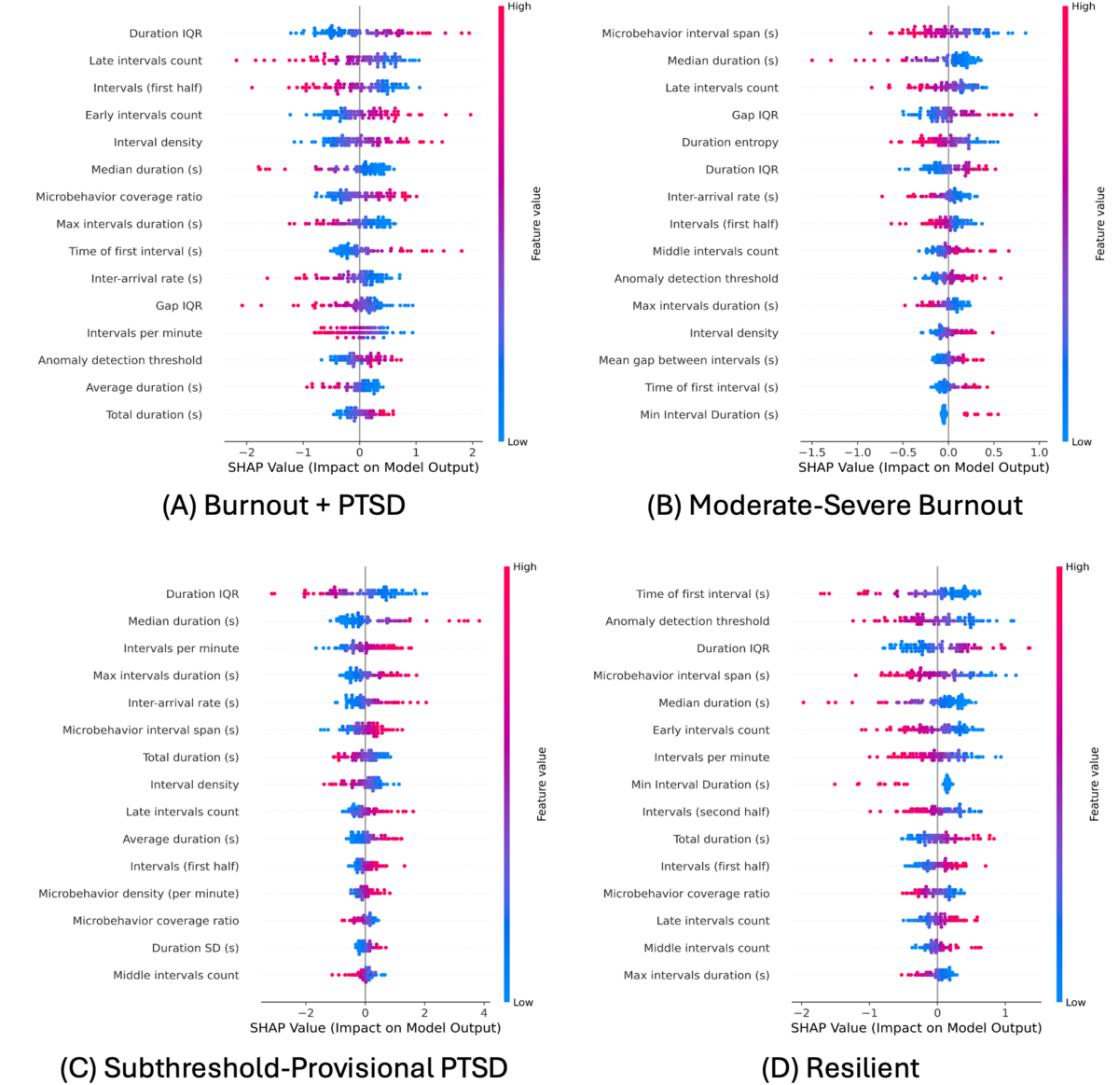
SHAP (Shapley additive explanations) summary plots illustrating the top features contributing to classification decisions for each psychological comorbidity profile. SHAP values represent the impact of each feature on model output. Color encodes feature magnitude (high=pink and low=blue). (A) Subthreshold-provisional posttraumatic stress disorder, (B) moderate-severe burnout, (C) resilient, and (D) moderate-severe burnout. PTSD: posttraumatic stress disorder.

Finally, we conducted an explainability analysis of the classification decisions using Shapley Additive Explanations (SHAP) [59], highlighting the most influential microbehavior interval features for each class (Figure 3).

## Results

### Overview

We analyzed 258 interview recordings from 151 HCWs. The average interview length was 16.08 (SD 1.72) minutes. Participants’ average age was 34.24 (SD 8.48) years, and the majority were female (n=93, 61.6%). Professional roles included registered nurses (n=54, 35.8%), resident physicians (n=47, 31.1%), faculty physicians (n=19, 12.6%), and other health care professionals (n=31, 20.5%), with an average of 4.96 (SD 5.70) years of clinical employment.

On the basis of our labeling procedure, the following are the sizes of our 4 symptom classes: “resilient”=121 (47%), “moderate-severe burnout”=54 (21%), “subthreshold-provisional PTSD”=31 (12%), and “burnout+PTSD”=52 (20%). Tables 1 and 2 summarize the demographics and clinical characteristics of the sample. Additional details about the sample are provided in Table S1 and Figure S1 in the Multimedia Appendix 1.

**Table 1:** Demographic characteristics of health care workers (N=151)a.

Characteristics		Values
Age (years), mean (SD)		34.24 (8.48)
**Sex at birth, n (%)**		
	Female	93 (61.59)
	Male	58 (38.41)
**Current position, n (%)**		
	Faculty physician	19 (12.58)
	Resident physician	47 (31.13)
	Registered nurse	54 (35.76)
	Other	31 (20.53)
Duration of employment (years), mean (SD)		4.96 (5.7)

^a^Continuous variables are presented as mean (SD) and categorical variables as n (%).

**Table 2:** Psychological symptoms scores of health care workers (N=151)^a^.

Clinical scores	Resilient group (n=121), mean (SD)	Moderate-severe burnout group (n=54), mean (SD)	Subthreshold-provisional PTSD^b^ group (n=31), mean (SD)	Burnout+PTSD group (n=52), mean (SD)
PCL-5^c^ total score	6.97 (6.34)	10.37 (7.01)	29.03 (7.71)	34.73 (8.66)
PCL-5 cluster B: re-experiencing	2.07 (2.56)	2.30 (2.07)	7.06 (3.74)	7.77 (3.71)
PCL-5 cluster C: avoidance	0.85 (1.25)	1.15 (1.42)	4.06 (2.00)	3.81 (2.11)
PCL-5 cluster D: negative mood and cognition	2.11 (2.50)	3.94 (3.59)	9.10 (3.93)	13.21 (4.73)
PCL-5 cluster E: hyperarousal	1.93 (2.05)	2.98 (2.84)	8.81 (4.21)	9.94 (3.71)
MBI-9^d^: emotional exhaustion	6.63 (3.70)	12.31 (3.12)	10.03 (3.40)	14.37 (2.21)
MBI-9: depersonalization	3.25 (2.66)	9.78 (2.73)	4.61 (3.16)	11.00 (3.82)
MBI-9: personal accomplishment	14.34 (2.93)	12.70 (2.90)	14.42 (2.46)	12.27 (3.09)

^a^Continuous variables are presented as mean (SD).

^b^PTSD: posttraumatic stress disorder.

^c^PCL-5: PTSD Checklist for Diagnostic and Statistical Manual, 5th Edition.

^d^MBI-9: Maslach Burnout Inventory General Survey 9-item.

### Microbehavior Intervals Characteristics

Across all recordings (N=258; 151 participants), the anomaly detection model (MOMENT) identified an average of 19.65 (SD 6.01) microbehavior intervals per interview (range 5-39). The average duration of these intervals was 1.31 (SD 1.10) seconds, ranging from 0.5-15.98 seconds. The first microbehavior interval occurred, on average, at 71.57 (SD 84.35) seconds into the interview, with the earliest detected at 0.68 seconds and the latest at 496.36 seconds. The distribution of key microbehavior interval characteristics is presented in Figure S2 of the Multimedia Appendix 1. See Tables S2-S4 in the Multimedia Appendix 1 for the detailed temporal features list.

### Classification Model Evaluation

For the hyperparameter search space and final configuration, refer to Table S5 in the Multimedia Appendix 1. Training performance results of the deep learning classifier are shown in Figure S3, illustrating its convergence behavior and macro– *F*_1_-score performance. To avoid overfitting, early stopping was implemented via Optuna’s “MedianPruner” during hyperparameter optimization. Within each cross-validation fold, the training data were split into internal training and validation subsets, and the cross-entropy loss was reported to Optuna at each epoch as an intermediate value. Trials were terminated early when the current trial’s validation loss was worse than the median of completed trials at the same epoch. Pruning decisions were based only on the internal validation data within each fold; the held-out fold was used exclusively for final evaluation.

Our classifier successfully predicted the 4 symptom classes on both the training and the test splits (Table S6 in the Multimedia Appendix 1). Weighted performance metrics on the test set were robust, with a precision of 0.81, recall of 0.79, accuracy of 78.3%, and a macro– *F*_1_-score of 0.75.

Figure 2 presents the model performance scores for the 4 symptom classes. The classifier demonstrated high discriminative ability for identifying participants in the “subthreshold-provisional PTSD” (area under the curve [AUC]=0.869), “moderate-severe burnout” (AUC=0.825) classes, and “burnout+PTSD” class (AUC=0.798). In contrast, identifying participants in the “resilient” class was less discriminatory, with a lower area under the receiver operating characteristic curve score of 0.686. Macroaverage and microaverage AUCs were 0.793 and 0.798, respectively, indicating consistent performance across classes and balanced treatment of minority labels.

Additionally, to assess the contribution of each modality, we conducted an ablation study by systematically removing 1 modality at a time from the complete multimodal framework. Excluding gaze or arousal-valence time-series signals resulted in the most significant performance drops, particularly in recall and *F*_1_-score. In contrast, 3D hands and body pose contributed less individually, although their removal still resulted in measurable degradation (Figure S4 in the Multimedia Appendix 1).

### Feature Importance Analysis

The SHAP analysis presented in Figure 3 visualizes the contribution of the top 15 most influential features per class, ranked by their average impact on the model’s output. Across all classes, features encoding variability (ie, duration interquartile and gap interquartile) and timing (ie, time of first interval, early intervals count, and late intervals count) frequently emerged as key drivers of model predictions.

For the “subthreshold-provisional PTSD” class, SHAP analysis revealed that the class was characterized by elevated variability and fragmentation in their behavioral dynamics, indicating frequent and short bursts of microbehavior intervals.

For the “moderate-severe burnout” class, the most influential features included an early onset of microbehavior intervals and prolonged durations of intervals. The model also highlighted measures of temporal irregularity and interval frequency, suggesting that this group was characterized by early activation and sustained, uneven patterns of microbehavior intervals across the interview.

Participants in the “burnout+PTSD” group showed distinctive patterns of both volatility and persistence in microbehavior intervals. The model was mostly influenced by high variability in microbehavior duration, as well as the timing and frequency of intervals across the interview. Both early and late interval counts, along with greater interval density and temporal dispersion, contributed strongly to classification. These findings suggest that individuals with combined burnout and PTSD display fragmented and irregular patterns of microbehavior intervals throughout the interview.

The SHAP analysis revealed a markedly different feature profile for the “resilient” class. These participants exhibited a more heterogeneous profile, characterized by fewer microbehavior intervals overall and less fluctuation in timing and duration compared to the symptomatic groups.

## Discussion

We present a novel multimodal framework for identifying psychological distress related to burnout and PTSD in HCWs by modeling brief, spontaneous nonverbal fluctuations, termed microbehaviors, as objective digital biomarkers from video-recorded interviews. By analyzing multimodal nonverbal signals from emotion-inducing interviews, we identified 4 clinically meaningful classes: “resilient,” “moderate-severe burnout,” “subthreshold-provisional PTSD,” and “burnout+PTSD.”

The classifier demonstrated strong overall discrimination, with particularly reliable separation of participants with PTSD symptoms, burnout symptoms, or a comorbid symptom profile. Temporal variability, irregularity, and onset of microbehaviors were the most informative predictors, highlighting distinct expressive profiles across classes. Ablation experiments further demonstrated the complementary contribution of modalities, underscoring the benefit of combining large-scale posture and movement signals (pose-derived gross motor activity) with subtle high-resolution cues (gaze dynamics and facial expression) for more accurate mental health assessment. Our findings underscore the potential to broaden the analytical focus from isolated facial microexpressions or narrative content to a wider spectrum of high-resolution, multimodal nonverbal behaviors.

Prior multimodal and behavioral screening studies using nonverbal signals have generally reported moderate-to-strong discrimination, most often in simpler binary case-control settings. For example, Stratou et al [33] demonstrated that automated nonverbal indicators can support prescreening for both depression and PTSD, with cross-validated *F*_1_-scores of approximately 0.71 for higher-severity classification, consistent with the challenges of subtle symptom detection from behavior alone. In a closely related trauma-exposed cohort, Schultebraucks et al [24] combined visual and auditory markers of arousal and mood and reported strong binary classification for PTSD (AUC=0.90; weighted *F*_1_-score=0.83) and depression (AUC=0.86; weighted *F*_1_-score=0.82), underscoring the potential of audiovisual markers while also highlighting how headline metrics shift with task formulation and label granularity. Complementing this, Stolicyn et al [25] showed that combining ecological face-tracking and eye-tracking features improved depression prediction, achieving a maximum accuracy of 79%, supporting our finding that gaze and affective or arousal cues contribute meaningfully to discrimination. In contrast, speech-only PTSD studies in curated binary datasets report very high AUC and accuracy [39], highlighting that direct comparison across modalities and study designs can vary substantially. Finally, the microexpression approach by Li et al [21] also achieved a high accuracy in controlled settings (accuracy ≈0.873) but relies on highly strict acquisition constraints than standard webcam interviews, motivating the practical value of our novel methodology in a real-world screening context. Taken together, our performance is broadly consistent with the range reported for scalable behavioral screening approaches, while extending prior work by emphasizing temporally localized microbehavior intervals and multiprofile differentiation rather than binary symptom detection.

Distinct microbehavior signatures were observed in both burnout and PTSD classes, thus supporting the hypothesis that dysregulated expressive dynamics reflect underlying psychological distress [60,61]. For example, we hypothesized that the fragmented and frequent microbehavior intervals in the “subthreshold-provisional PTSD” group may indicate heightened physiological reactivity and unstable emotional regulation, mirroring hallmark PTSD symptoms such as hyperarousal and intrusive re-experiencing [62], where individuals repeatedly relive trauma-related cues through abrupt, involuntary responses. The early onset and prolonged microbehavior intervals in the “moderate-severe burnout” group may suggest a diminished capacity to regulate or recover from affective activation. This could potentially align with sustained emotional exhaustion and blunted affective engagement, which are characteristic of severe burnout [63]. The combined “burnout+PTSD” group exhibited volatility and wide dispersion of microbehavior intervals across the interview. We postulate that this pattern could be consistent with the emotional exhaustion of burnout [63], coupled with the reactive instability and heightened sensitivity to triggers typical of PTSD [62].

Conversely, classification performance was lower for the “resilient” group. SHAP analysis revealed that these individuals exhibited fewer distinctive microbehavior patterns, which may account for the reduced discriminative accuracy. Rather than exhibiting a consistent temporal signature, their SHAP distribution suggested heterogeneous dynamics, reflecting the multifaceted nature of resilience. This aligns with prior research showing that resilience is not defined solely by the absence of arousal- or mood-related markers, and is often harder to discriminate than symptomatic patients [64]. As such, nonverbal behavioral signals alone may not fully capture resilience, underscoring the need for an even more comprehensive methodological approach. Practically, these findings position our framework as a screening tool for identifying elevated distress rather than a tool to “confirm” resilience.

From a clinical perspective, these findings highlight the potential of microbehavior analysis as a scalable and unobtrusive complement to standard clinical interviews and psychometric questionnaires. Our framework operates without manual annotation, is language-agnostic, and requires only short, semistructured interviews, making it particularly suitable for frontline professionals who may minimize or suppress verbal disclosure of distress [65]. Importantly, the use of anomaly-based modeling enables the detection of behavior deviations that are imperceptible to the human eye, providing clinicians with objective cues that may precede or corroborate self-reported symptoms. Nevertheless, as with any digital mental health screening approach, ethical considerations (eg, data privacy and algorithmic bias) must also be addressed when deploying such systems in real-world settings.

Beyond classification, the ability to analyze multimodal signals at high temporal resolution makes this framework a potential tool for discovering latent behavioral phenotypes across various mental health conditions. Future work should extend these findings by clarifying which microbehavioral dynamics map onto specific symptom domains, how they evolve over time, and how they manifest in comorbid presentations.

Several limitations warrant consideration, many of which reflect deliberate design trade-offs aimed at prioritizing ecological validity and feasibility within realistic telehealth constraints. First, our cohort consisted exclusively of HCWs. This increases the ecological validity in this population and aligns with our intended screening use case but may also limit generalizability to other trauma-exposed groups such as military personnel or first responders. Second, the framework is sensitive to video quality and recording conditions that vary substantially in real-world telehealth settings (eg, camera angle, motion blur, occlusions, and intermittent connectivity). These factors can introduce noise and missingness of different modalities and may affect the stability of anomaly scores and the resulting interval segmentation. Although our pipeline retains such frames and propagates missing values to downstream aggregation, performance and interpretability are expected to degrade when visibility is poor, or tracking failures are frequent; future work should quantify quality thresholds and incorporate explicit quality-aware confidence measures. Third, several pipeline components rely on fixed preprocessing and segmentation choices. These parameters were chosen based on empirical inspection to reduce overfragmentation and to robustly handle transient tracking noise. Still, they can influence the number, duration, and temporal distribution of detected intervals, thereby affecting derived interval-level statistics. Although the central conclusion that temporally localized expressive episodes contain a discriminative signal is supported by the overall approach, future studies should validate parameter sensitivity across settings and explore principled calibration strategies that improve cross-recording comparability. Fourth, our classification operates on aggregated interval-level features. Although this design choice improves interpretability and scalability, it may discard within-interval temporal structure and assume that summary statistics sufficiently capture clinically relevant dynamics. Additionally, although extracting features from aggregated microbehavior intervals emphasizes group-level regularities, they may also limit personalization.

Finally, we intentionally focused on nonverbal modalities to reduce linguistic and fluency-related biases and to target a broadly deployable telehealth workflow; however, this decision may restrict the interpretability and reliability of the detected patterns. Incorporating questions timing and the semantic content of the participant’s response could potentially enhance the diagnostic relevance of these patterns.

In conclusion, this study introduces a multimodal, anomaly-based framework for identifying psychological distress in HCWs through the analysis of microbehaviors—brief, involuntary fluctuations in nonverbal expressions. By providing objective, annotation-free biomarkers that are sensitive to subtle behavioral changes, the framework holds promise as a complementary tool that enhances traditional mental health assessments.

## Appendix

Multimedia Appendix 1 Additional details about the sample.

## Data Availability

All requests for raw and analyzed data, as well as related materials, will be reviewed by our legal department at New York University Grossman School of Medicine to verify whether the request is subject to any intellectual property or confidentiality constraints. Any data and materials that can be shared will be released via a material transfer agreement for noncommercial research purposes. The request should be addressed to KS. All requests for programming code should be addressed to KS. Any materials that can be shared will be released via a material transfer agreement for noncommercial research purposes.

## Abbreviations

AUC: area under the curve
HCW: health care worker
HIPAA: Health Insurance Portability and Accountability Act
MOMENT: a Family of Open Time-Series Foundation Models
PTSD: posttraumatic stress disorder
SHAP: Shapley Additive Explanations

## References

[1] Pappa S, Ntella V, Giannakas T, Giannakoulis VG, Papoutsi E, Katsaounou P (2020). Prevalence of depression, anxiety, and insomnia among healthcare workers during the COVID-19 pandemic: a systematic review and meta-analysis. Brain Behav Immun.

[2] Fear NT, Jones M, Murphy D, Hull L, Iversen AC, Coker B, Machell L, Sundin J, Woodhead C, Jones N, Greenberg N, Landau S, Dandeker C, Rona RJ, Hotopf M, Wessely S (2010). What are the consequences of deployment to Iraq and Afghanistan on the mental health of the UK armed forces? A cohort study. Lancet.

[3] Haugen PT, McCrillis AM, Smid GE, Nijdam MJ (2017). Mental health stigma and barriers to mental health care for first responders: a systematic review and meta-analysis. J Psychiatr Res.

[4] Norman SB, Feingold JH, Kaye-Kauderer H, Kaplan CA, Hurtado A, Kachadourian L, Feder A, Murrough JW, Charney D, Southwick SM, Ripp J, Peccoralo L, Pietrzak RH (2021). Moral distress in frontline healthcare workers in the initial epicenter of the COVID-19 pandemic in the United States: relationship to PTSD symptoms, burnout, and psychosocial functioning. Depress Anxiety.

[5] Cieslak R, Shoji K, Douglas A, Melville E, Luszczynska A, Benight CC (2014). A meta-analysis of the relationship between job burnout and secondary traumatic stress among workers with indirect exposure to trauma. Psychol Serv.

[6] Mealer M, Burnham EL, Goode CJ, Rothbaum B, Moss M (2009). The prevalence and impact of post traumatic stress disorder and burnout syndrome in nurses. Depress Anxiety.

[7] Maslach C, Schaufeli WB, Leiter MP (2001). Job burnout. Annu Rev Psychol.

[8] Woo T, Ho R, Tang A, Tam W (2020). Global prevalence of burnout symptoms among nurses: a systematic review and meta-analysis. J Psychiatr Res.

[9] Shen X, Xu H, Feng J, Ye J, Lu Z, Gan Y (2022). The global prevalence of burnout among general practitioners: a systematic review and meta-analysis. Fam Pract.

[10] Chirico F, Afolabi AA, Ilesanmi OS, Nucera G, Ferrari G, Sacco A, Szarpak L, Crescenzo P, Magnavita N, Leiter M (2021). Prevalence, risk factors and prevention of burnout syndrome among healthcare workers: an umbrella review of systematic reviews and meta-analyses. J Health Soc Sci.

[11] Shalev A, Liberzon I, Marmar C (2017). Post-traumatic stress disorder. N Engl J Med.

[12] D'Ettorre G, Pellicani V, Ceccarelli G (2020). Post-traumatic stress disorder symptoms in healthcare workers: a ten-year systematic review. Acta Biomed.

[13] Hill JE, Harris C, Danielle L C, Boland P, Doherty AJ, Benedetto V, Gita BE, Clegg AJ (2022). The prevalence of mental health conditions in healthcare workers during and after a pandemic: systematic review and meta-analysis. J Adv Nurs.

[14] Greene-Shortridge TM, Britt TW, Castro CA (2007). The stigma of mental health problems in the military. Mil Med.

[15] Gold KJ, Andrew LB, Goldman EB, Schwenk TL (2016). "I would never want to have a mental health diagnosis on my record": a survey of female physicians on mental health diagnosis, treatment, and reporting. Gen Hosp Psychiatry.

[16] Chen X, Luo T (2023). Catching elusive depression via facial micro-expression recognition. IEEE Commun Mag.

[17] Ekman P, Friesen WV (1969). Nonverbal leakage and clues to deception. Psychiatry.

[18] Ekman P (2003). Emotions Revealed: Recognizing Faces and Feelings to Improve Communication and Emotional Life.

[19] Pfister T, Li X, Zhao G, Pietikainen M (2011). Recognising spontaneous facial micro-expressions. Proceedings of the 2011 International Conference on Computer Vision.

[20] Zhao G, Li X, Li Y, Pietikäinen M (2023). Facial micro-expressions: an overview. Proc IEEE.

[21] Li X, Yi X, Ye J, Zheng Y, Wang Q (2024). SFTNet: a microexpression-based method for depression detection. Comput Methods Programs Biomed.

[22] Shatte AB, Hutchinson DM, Teague SJ (2019). Machine learning in mental health: a scoping review of methods and applications. Psychol Med.

[23] Zhernova P, Bodyanskiy Y, Yatsenko B, Zavgorodnii I (2020). Detection and prevention of professional burnout using machine learning methods. Proceedings of the 2020 IEEE 15th International Conference on Advanced Trends in Radioelectronics, Telecommunications and Computer Engineering.

[24] Schultebraucks K, Yadav V, Shalev AY, Bonanno GA, Galatzer-Levy IR (2020). Deep learning-based classification of posttraumatic stress disorder and depression following trauma utilizing visual and auditory markers of arousal and mood. Psychol Med.

[25] Stolicyn A, Steele JD, Seriès P (2020). Prediction of depression symptoms in individual subjects with face and eye movement tracking. Psychol Med.

[26] Ding Z, Wang Z, Zhang Y, Cao Y, Liu Y, Shen X, Tian Y, Dai J (2025). Trade-offs between machine learning and deep learning for mental illness detection on social media. Sci Rep.

[27] Yan WJ, Wu Q, Liang J, Chen YH, Fu X (2013). How fast are the leaked facial expressions: the duration of micro-expressions. J Nonverbal Behav.

[28] Singh NN, McKay JD, Singh AN (1998). Culture and mental health: nonverbal communication. J Child Fam Stud.

[29] Tarik B, Ashour S, Gomaa W (2024). Mental health assessment: analyzing body language patterns and emotional expression. Proceedings of the 2024 Intelligent Methods, Systems, and Applications.

[30] Ringwald WR, Feltman S, Schwartz HA, Samaras D, Khudari C, Luft BJ, Kotov R (2025). Day-to-day dynamics of facial emotion expressions in posttraumatic stress disorder. J Affect Disord.

[31] Coll SY, Eustache F, Doidy F, Fraisse F, Peschanski D, Dayan J, Gagnepain P, Laisney M (2022). Avoidance behaviour generalizes to eye processing in posttraumatic stress disorder. Eur J Psychotraumatol.

[32] Bianchi R, Laurent E (2015). Emotional information processing in depression and burnout: an eye-tracking study. Eur Arch Psychiatry Clin Neurosci.

[33] Stratou G, Scherer S, Gratch J, Morency LP (2013). Automatic nonverbal behavior indicators of depression and PTSD: exploring gender differences. Proceedings of the 2013 Humaine Association Conference on Affective Computing and Intelligent Interaction.

[34] Mitani S, Fujita M, Nakata K, Shirakawa T (2006). Impact of post-traumatic stress disorder and job-related stress on burnout: a study of fire service workers. J Emerg Med.

[35] Restauri N, Sheridan AD (2020). Burnout and posttraumatic stress disorder in the coronavirus disease 2019 (COVID-19) pandemic: intersection, impact, and interventions. J Am Coll Radiol.

[36] Blázquez-García A, Conde A, Mori U, Lozano JA (2021). A review on outlier/anomaly detection in time series data. ACM Comput Surv.

[37] Raman N, Cao M, Tsvetkov Y, Kästner C, Vasilescu B (2020). Stress and burnout in open source: toward finding, understanding, and mitigating unhealthy interactions. Proceedings of the ACM/IEEE 42nd International Conference on Software Engineering: New Ideas and Emerging Results.

[38] Baxter SL, Saseendrakumar BR, Cheung M, Savides TJ, Longhurst CA, Sinsky CA, Millen M, Tai-Seale M (2022). Association of electronic health record inbasket message characteristics with physician burnout. JAMA Netw Open.

[39] Hu J, Zhao C, Shi C, Zhao Z, Ren Z (2024). Speech-based recognition and estimating severity of PTSD using machine learning. J Affect Disord.

[40] Cummins N, Scherer S, Krajewski J, Schnieder S, Epps J, Quatieri TF (2015). A review of depression and suicide risk assessment using speech analysis. Speech Commun.

[41] Kring AM, Stuart BK, Harrigan JA, Rosenthal R, Scherer KR (2005). Nonverbal behavior and psychopathology. The New Handbook of Methods in Nonverbal Behavior Research.

[42] Lavelle M, Healey PG, McCabe R (2014). Nonverbal behavior during face-to-face social interaction in schizophrenia: a review. J Nerv Ment Dis.

[43] Berry DS, Pennebaker JW (1993). Nonverbal and verbal emotional expression and health. Psychother Psychosom.

[44] Code of Federal Regulations.

[45] Leiter MP, Shaughnessy K (2006). The areas of worklife model of burnout: tests of mediation relationships. Ergonomia.

[46] Rotenstein LS, Torre M, Ramos MA, Rosales RC, Guille C, Sen S, Mata DA (2018). Prevalence of burnout among physicians: a systematic review. JAMA.

[47] Blevins CA, Weathers FW, Davis MT, Witte TK, Domino JL (2015). The Posttraumatic Stress Disorder Checklist for DSM-5 (PCL-5): development and initial psychometric evaluation. J Trauma Stress.

[48] Klein AB, Schnurr PP, Bovin MJ, Friedman MJ, Keane TM, Marx BP (2024). An empirical investigation of definitions of subthreshold posttraumatic stress disorder. J Trauma Stress.

[49] Biscoe N, Campbell GM, Murphy D (2025). Beyond the threshold: considering and exploring subthreshold PTSD and co-morbid mental health in a clinical military sample. Eur J Trauma Dissoc.

[50] No authors listed (2024). TRIPOD+AI statement: updated guidance for reporting clinical prediction models that use regression or machine learning methods. BMJ.

[51] Collins GS, Dhiman P, Andaur Navarro CL, Ma J, Hooft L, Reitsma JB, Logullo P, Beam AL, Peng L, Van Calster B, van Smeden M, Riley RD, Moons KG (2021). Protocol for development of a reporting guideline (TRIPOD-AI) and risk of bias tool (PROBAST-AI) for diagnostic and prognostic prediction model studies based on artificial intelligence. BMJ Open.

[52] Facetorch. GitHub.

[53] Ververas E, Gkagkos P, Deng J, Doukas MC, Guo J, Zafeiriou S (2024). 3DGazeNet: generalizing 3D gaze estimation with weak-supervision from synthetic views. Proceedings of the 18th European Conference on Computer Vision.

[54] Dwivedi S, Sun Y, Patel P, Feng Y, Black MJ (2024). TokenHMR: advancing human mesh recovery with a tokenized pose representation. Proceedings of the 2024 IEEE/CVF Conference on Computer Vision and Pattern Recognition.

[55] Potamias RA, Zhang J, Deng J, Zafeiriou S (2025). WiLoR: end-to-end 3D hand localization and reconstruction in-the-wild. Proceedings of the 2025 IEEE/CVF Conference on Computer Vision and Pattern Recognition.

[56] Goswami M, Szafer K, Choudhry A, Cai Y, Li S, Dubrawski A (2024). MOMENT: a family of open time-series foundation models. Proceedings of the 41st International Conference on Machine Learning.

[57] Akiba T, Sano S, Yanase T, Ohta T, Koyama M (2019). Optuna: a next-generation hyperparameter optimization framework. Proceedings of the 25th ACM SIGKDD International Conference on Knowledge Discovery & Data Mining.

[58] Chawla NV, Bowyer KW, Hall LO, Kegelmeyer WP (2002). SMOTE: synthetic minority over-sampling technique. J Artif Intell Res.

[59] Lundberg SM, Lee SI (2017). A unified approach to interpreting model predictions. Proceedings of the 31st International Conference on Neural Information Processing Systems.

[60] Cole PM, Michel MK, Teti LO (1994). The development of emotion regulation and dysregulation: a clinical perspective. Monogr Soc Res Child Dev.

[61] D'Agostino A, Covanti S, Rossi Monti M, Starcevic V (2017). Reconsidering emotion dysregulation. Psychiatr Q.

[62] Yufik T, Simms LJ (2010). A meta-analytic investigation of the structure of posttraumatic stress disorder symptoms. J Abnorm Psychol.

[63] Maslach C, Leiter MP, Fink G (2016). Burnout. Stress: Concepts, Cognition, Emotion, and Behavior.

[64] Schultebraucks K, Choi KW, Galatzer-Levy IR, Bonanno GA (2021). Discriminating heterogeneous trajectories of resilience and depression after major life stressors using polygenic scores. JAMA Psychiatry.

[65] Waegemakers Schiff J, Lane AM (2019). PTSD symptoms, vicarious traumatization, and burnout in front line workers in the homeless sector. Community Ment Health J.

