# Supplementary material for: Detection of Microbehavior Intervals for Predicting Mental Health: Clinically Relevant and Advanced Multimodal Temporal Analysis
Source: J Med Internet Res. 2026 May 27;28:e87049. doi: 10.2196/87049 (PMC13254507; doi:10.2196/87049)
Supplement: Multimedia Appendix 1 [file jmir_v28i1e87049_app1.docx]

**Detection of Micro-Behavior Intervals: A Clinically Relevant and Advanced Multimodal Temporal Approach for Predicting Mental Health**

Sapir Gershov^1^, Charlotte E. Hilberdink^1^, Yiwen Zhao^1^, Sarah B. Birnbaum^1^, Victoria Mueller^1,2^, Stephen P. Wall^3,4^, Katharina Schultebraucks^1,4*^

^1^ Department of Psychiatry, NYU Grossman School of Medicine, New York, NY, USA.

^2^ Department of Biomedical Engineering, NYU Tandon School of Engineering, New York, NY, USA.
^3^ Department of Emergency Medicine, NYU Grossman School of Medicine, New York, NY, USA.

^4^ Department of Population Health, NYU Grossman School of Medicine, New York, NY, USA.

*Corresponding author. Email:

[Katharina.Schultebraucks@nyulangone.org](mailto:Katharina.Schultebraucks@nyulangone.org)

**Supplementary Material**

| **Table S1: Demographic characteristics and psychological symptoms of HCWs.** | | | | | | | | | |
| --- | --- | --- | --- | --- | --- | --- | --- | --- | --- |
| **Demographics (N=151)** | | | | | | | | | |
| Age | 34.24 (8.48) | | | | | | | | |
| Sex at Birth | Female – 93 (61.59%) | | | | | Male – 58 (38.41%) | | | |
| Race | White – 70 (46.36%) | | Black - 23 (15.23%) | | | Asian – 37 (24.50%) | | | Other – 21 (13.91%) |
| Ethnicity | Hispanic – 21 (13.91%) | | | | Not Hispanic – 125 (82.78%) | | | Other – 5 (3.31%) | |
| Highest Education | College Grad – 48 (31.79%) | | | | Grad School – 95 (62.91%) | | | Other – 8 (5.30%) | |
| Current Position | Faculty Physician – 19 (12.58%) | | Resident Physician – 47 (31.13%) | | | Registered Nurse – 54 (35.76%) | | | Other – 31 (20.53%) |
| Duration of Employment (years) | 4.96 (5.70) | | | | | | | | |
| **Clinical Scores (N=258)** | | | | | | | | | |
| Symptoms Severity | | ‘Resilient’  n=121 | | ‘M-S Burnout’  n=54 | | | ‘S-P PTSD’  n=31 | | ‘Burnout + PTSD’  n=52 |
| PCL-5 total score | | 6.97 (6.34) | | 10.37 (7.01) | | | 29.03 (7.71) | | 34.73 (8.66) |
| PCL-5, B | | 2.07 (2.56) | | 2.30 (2.07) | | | 7.06 (3.74) | | 7.77 (3.71) |
| PCL-5, C | | 0.85 (1.25) | | 1.15 (1.42) | | | 4.06 (2.00) | | 3.81 (2.11) |
| PCL-5, D | | 2.11 (2.50) | | 3.94 (3.59) | | | 9.10 (3.93) | | 13.21 (4.73) |
| PCL-5, E | | 1.93 (2.05) | | 2.98 (2.84) | | | 8.81 (4.21) | | 9.94 (3.71) |
| MBI-9, EE | | 6.63 (3.70) | | 12.31 (3.12) | | | 10.03 (3.40) | | 14.37 (2.21) |
| MBI-9, DEP | | 3.25 (2.66) | | 9.78 (2.73) | | | 4.61 (3.16) | | 11.00 (3.82) |
| MBI-9, PA | | 14.34 (2.93) | | 12.70 (2.90) | | | 14.42 (2.46) | | 12.27 (3.09) |

Continuous variables are presented as mean (SD) and categorical variables are presented as n (%).

M-S = Moderate-Severe; S-P = Subthreshold-Provisional.

PCL-5, B: Re-experiencing, PCL-5, C: Avoidance, PCL-5, D: Negative Mood/Cognition, PCL-5, E: Hyperarousal.
MBI-9, EE: Emotional Exhaustion, MBI-9, DEP: Depersonalization, MBI-9, PA: Personal Accomplishment.

**Table S2: *Microbehavior* interval features descriptions and interpretations (part 1 of 3).**

| **Variable** | **Description** | **Interpretation** |
| --- | --- | --- |
| Anomaly detection threshold | 95th percentile threshold used to define micro-behavior intervals. | Threshold score above which time points are flagged as anomalous; determines sensitivity. |
| Total microbehavior intervals | Total number of micro-behavior intervals above threshold. | Measures how many expressive bursts were detected; may reflect behavioral instability. |
| Intervals per minute | Frequency of micro-behavior intervals per minute. | Frequency of bursts per minute; a proxy for reactivity or agitation. |
| Total duration | Cumulative duration of all microbehavior intervals. | Total time spent in expressive states; may suggest sustained arousal or burden. |
| Average duration | Average length of a microbehavior interval. | Typical interval duration; contextual measure of expressivity length. |
| Median duration | Median duration of all microbehavior intervals. | Middle value of interval durations; less sensitive to outliers than mean. |
| Duration SD | Standard deviation of microbehavior interval durations. | Spread of duration values; high variance may indicate dysregulation. |
| Duration variance | Variance of microbehavior interval durations. | Measures dispersion in durations across intervals. |
| Max interval duration | Duration of the longest microbehavior interval. | Length of the longest burst; extreme values may reflect breakdowns in regulation. |
| Min interval duration | Duration of the shortest microbehavior interval. | Shortest interval detected; may reflect brief reactivity spikes. |
| Interval density | Proportion of video duration spent in microbehavior activity. | Proportion of the video occupied by micro-behavior intervals. |
| Time of first interval | Timestamp of the beginning of the first microbehavior interval. | Onset of first expressive burst; earlier start may reflect low inhibition. |
| Mean gap between intervals | Mean time between the end of one microbehavior interval and the start of the next. | Average time between intervals; inverse of density, reflects spacing. |
| Gap variance | Variance of gap lengths between microbehavior intervals. | Variation in spacing; irregular gaps can indicate fragmentation. |
| Max gap between intervals | Longest time gap between any two consecutive microbehavior intervals. | Longest time without detected expressivity; reflects disengagement. |
| Early intervals count | Number of microbehavior intervals in the first third of the video. | Expressive activity early in the video; may reflect immediate engagement or distress. |

**Table S3: *Micro-behavior* interval features descriptions and interpretations (part 2 of 3).**

| **Variable** | **Description** | **Interpretation** |
| --- | --- | --- |
| Intervals middle count | Number of microbehavior intervals in the middle third of the video. | Activity during middle segment; may indicate sustained engagement. |
| Late intervals count | Number of microbehavior intervals in the final third of the video. | Late-phase expressivity; can reflect fatigue, avoidance, or cumulative arousal. |
| Inter-arrival rate | Average time between the start of consecutive microbehavior intervals. | Average time between starts of intervals; temporal regularity metric. |
| Microbehavior interval span | Time range between the first and last microbehavior interval (not total coverage). | Time range from first to last interval; overall temporal spread of expressivity. |
| Microbehavior density (per min) | Number of microbehavior intervals per minute within their span. | Density of expressivity relative to its own temporal span; compactness measure. |
| Microbehavior coverage ratio | Fraction of the full video duration covered by microbehavior intervals. | Proportion of the video time covered by intervals; alternative density measure. |
| Duration IQR | Interquartile range of microbehavior interval durations — measure of spread. | IQR of durations; robustness measure of temporal expressivity variability. |
| Gap IQR | Interquartile range of gaps between microbehavior intervals. | IQR of gaps; variability in timing between bursts. |
| Gaps-to-durations ratio | Mean gap length divided by mean microbehavior interval length. | Ratio of inactivity to activity; interpretable as temporal modulation efficiency. |
| Duration entropy | Entropy (disorder) of the microbehavior interval duration distribution. | Unpredictability in durations; reflects affective volatility. |
| Duration trend slope | Linear slope of microbehavior interval durations over time (positive = increasing). | Temporal slope of duration trend; increasing or decreasing control over time. |
| Intervals in first half | Number of microbehavior intervals in the first half of the video | Total intervals in first half; early video behavioral engagement. |
| Intervals in second half | Number of microbehavior intervals in the second half of the video. | Total intervals in second half; late engagement or symptom emergence. |

**Table S4: Micro-behavior interval features descriptions and interpretations (part 3 of 3).**

| **Variable** | **Description** | **Interpretation** |
| --- | --- | --- |
| Duration slope sign change | Number of sign changes in the linear slope of interval durations. | Measures how frequently the direction of expressivity trend reverses; high values reflect unstable modulation. |
| Burstiness index | Ratio of standard deviation to mean of inter-arrival times between micro-behavior intervals. | Captures irregularity in expressive behavior; higher values indicate behavioral unpredictability or volatility. |
| Symmetry index | Absolute difference between number of intervals in first and second half, normalized by total intervals. | Reflects temporal balance of expressivity; high asymmetry may indicate escalating emotional effort. |
| CV duration | Coefficient of variation of interval durations (std/mean). | Normalized measure of expressivity variability; robust to absolute scale. |
| Initial latency (s) | Time from video start to the onset of first micro-behavior interval. | Captures emotional inhibition or delayed activation; shorter latency may indicate impulsivity or lowered regulation. |
| Last interval end (s) | Timestamp of the last interval's end. | Indicates the temporal endpoint of expressive engagement; longer durations may reflect persistent arousal. |
| High frequence ratio | Proportion of intervals shorter than the median duration. | Estimates jittery expressivity; higher values suggest fragmented or dysregulated affective behavior. |

**Table S5: Hyperparameter search space and final selected values.**

| **Hyperparameter** | **Search Space** | **Final Value** |
| --- | --- | --- |
| Number of layers | {2, 3, 4, 5} | 4 |
| Units in Layer 0 | [32, 256] | 75 |
| Units in Layer 1 | [32, 256] | 52 |
| Units in Layer 2 | [32, 256] | 40 |
| Units in Layer 3 | [32, 256] | 42 |
| Units in Layer 4 | [32, 256] | N/A |
| Activation function | {ReLU, Leaky ReLU, GELU, Sigmoid, Tanh} | Tanh |
| Dropout rate | [0.0, 0.5] | 0.172 |
| Normalization type | {None, BatchNorm, LayerNorm} | LayerNorm |
| Optimizer | {Adam, AdamW, RMSProp, SGD} | AdamW |
| Learning rate | [0.0001, 0.01] | 0.00245 |
| Weight decay | [0.00001, 0.001] | 0.000156 |
| Batch size | {32, 64, 128} | 64 |

**Table S6: Classification model weighted performance metrics on the train (averaged) and test sets.**

|  | **Accuracy** | **Precision** | **Recall** | **F_1_-score** |
| --- | --- | --- | --- | --- |
| **Train set** | $0.861\pm0.059$ | $0.874\pm0.033$ | $0.801\pm0.127$ | $0.809\pm0.061$ |
| **Test set** | $0.783$ | $0.814$ | $0.788$ | $0.750$ |

| **Figure S1: Distribution of PTSD symptom severity and burnout subscale scores. The boxplot shows the PCL-5 symptom clusters (Cluster B: Re-experiencing, Cluster C: Avoidance, Cluster D: Negative Mood/Cognition, Cluster E: Hyperarousal) and total PCL-5 score, along with the three subscales of the Maslach Burnout Inventory (EE: Emotional Exhaustion, DEP: Depersonalization, PA: Personal Accomplishment).** | |
| --- | --- |
| 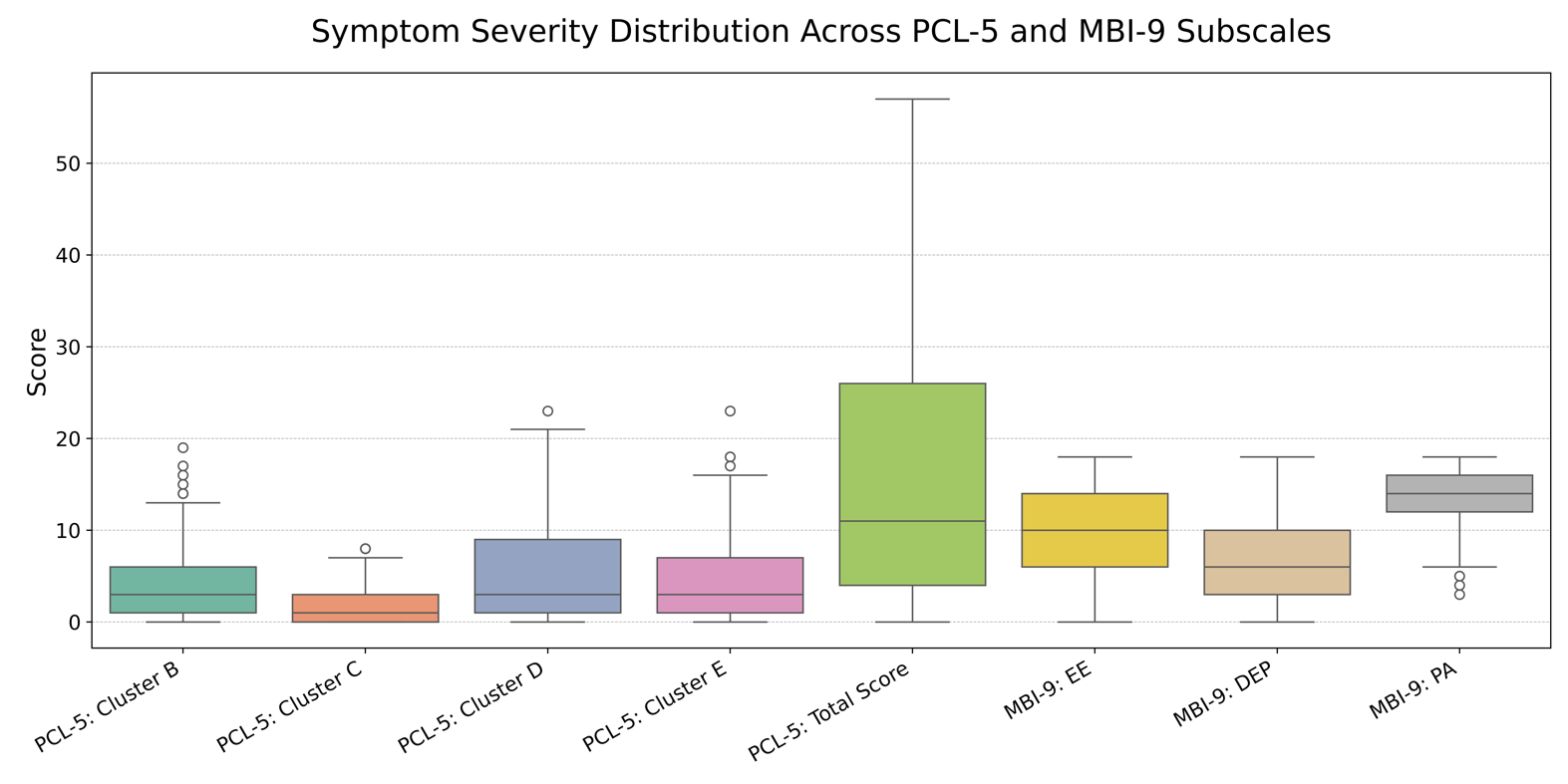 |  |

| **Figure S2: Boxplots of key interval features by comorbidity class (Resilient, Moderate-Severe Burnout, Subthreshold-Provisional PTSD, Burnout + PTSD). Group sizes are shown on the x-axis.** | |
| --- | --- |
| 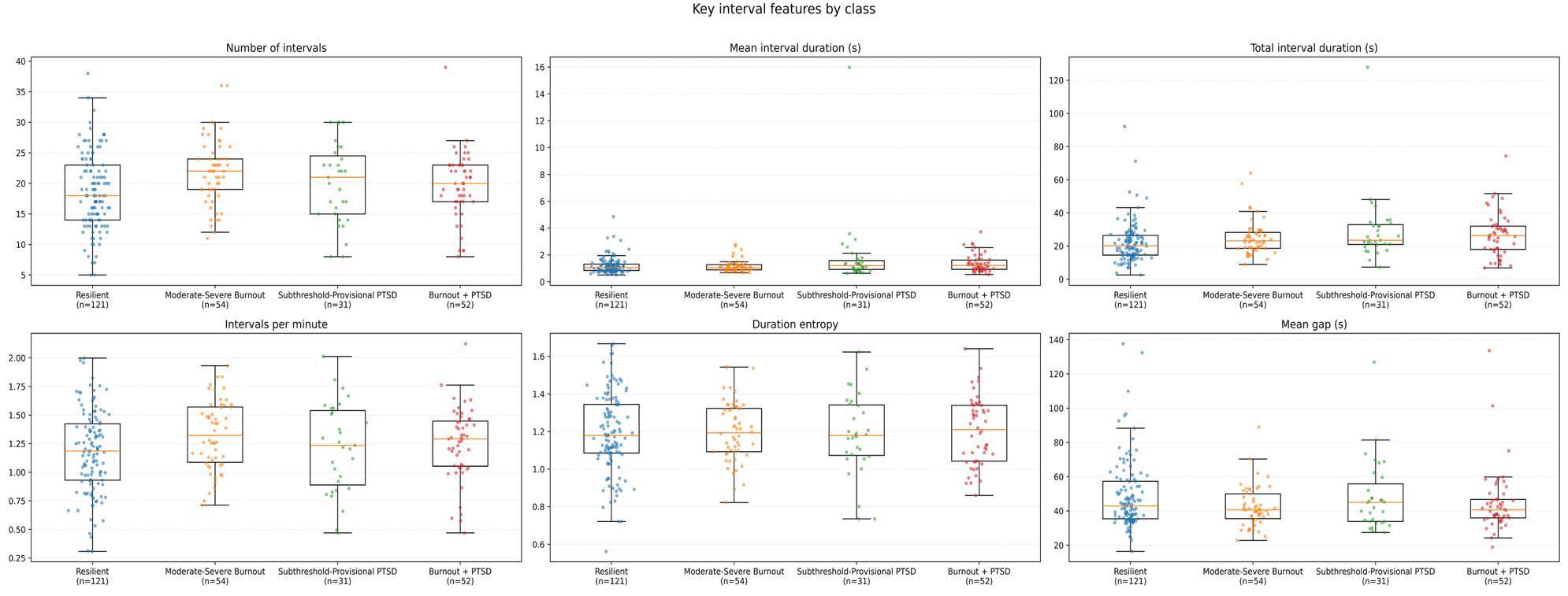 |  |

| **Figure S3: Training and validation dynamics of the deep learning classifier.**  Left: Training and validation loss curves across epochs, with early stopping applied at epoch 203. Right: Validation macro-F1 score across epochs. The best macro-F1 was achieved at epoch 199 (0.816), closely preceding the early stopping point. |
| --- |
| 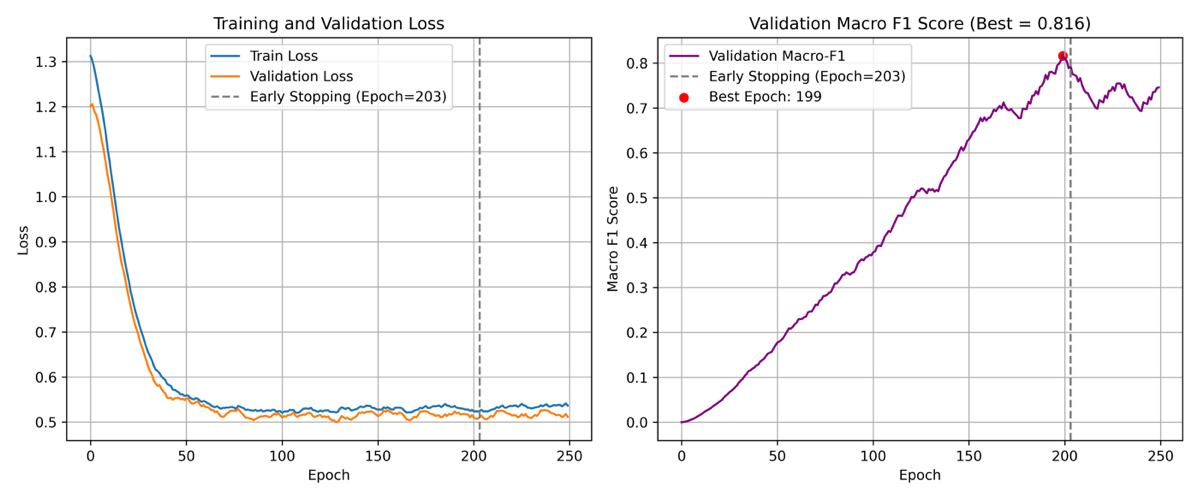 |

| **Figure S4:** **Ablation study results.** Radar plot depicting the results of an ablation study across five evaluation metrics (Accuracy, Precision, Recall, F1 score, and Macro AUROC). Each line represents model performance when one modality is removed from the full model (Combined Model). The full model integrates six input modalities: 3D gaze, facial action units, arousal-valence, 3D head pose, 3D hand pose, and 3D body pose. |
| --- |
| 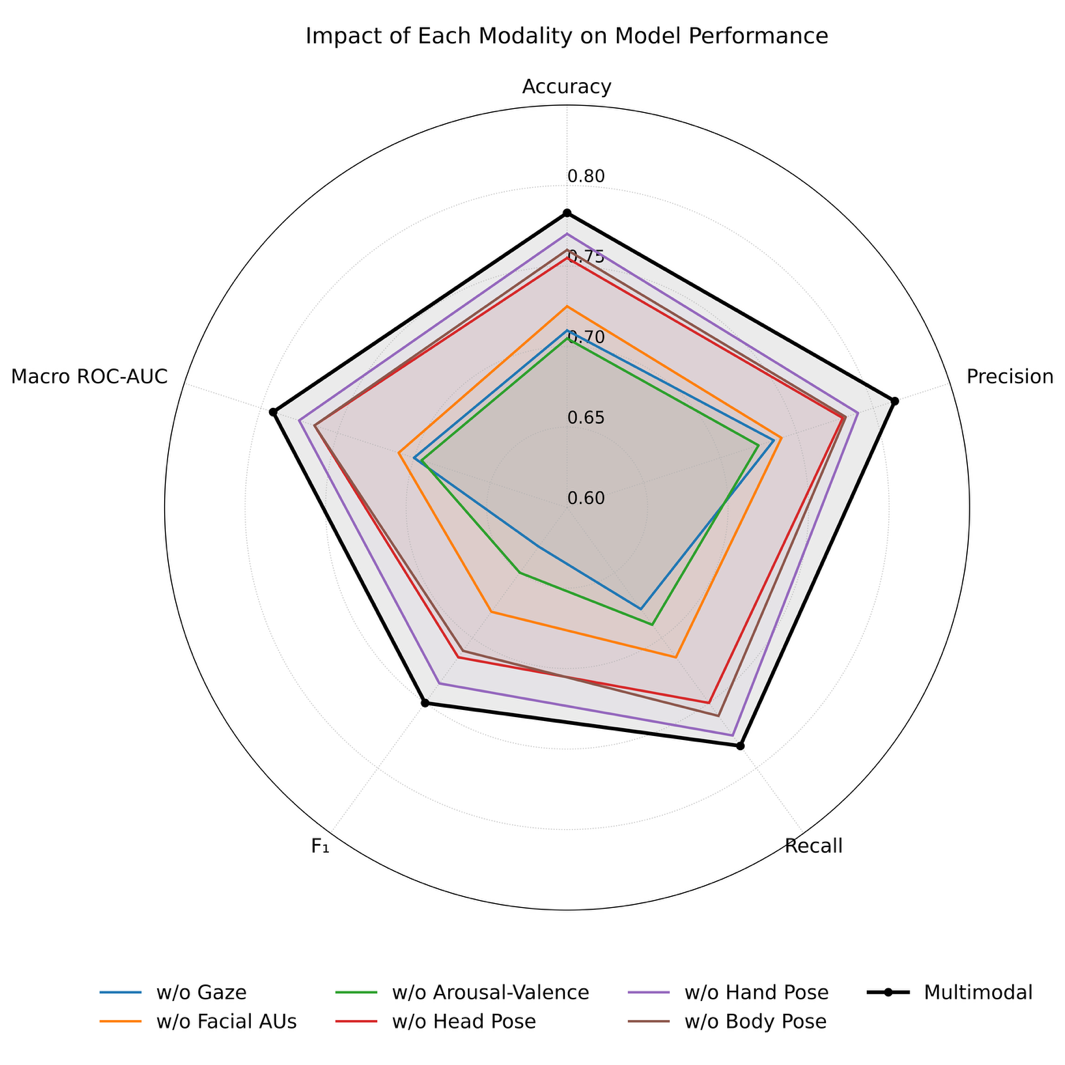 |

**Semi-structured interview questions:**

1. “Tell me about a case that you were involved with in the ED where you felt there was an unexpected and bad patient outcome.”
2. “Tell me how the patient outcome made you feel as the responsible provider.”
3. “Has this patient case changed your view of yourself as a clinician and how you approach patient care?”
4. “Tell me about a situation where you asked yourself, am I given enough recognition for my efforts?”
5. “What are your expectations about your role and your work as an ED clinician in the future?”
